# Age-Related Retinopathy in NRF2-Deficient Mice

**DOI:** 10.1371/journal.pone.0019456

**Published:** 2011-04-29

**Authors:** Zhenyang Zhao, Yan Chen, Jian Wang, Paul Sternberg, Michael L. Freeman, Hans E. Grossniklaus, Jiyang Cai

**Affiliations:** 1 Vanderbilt Eye Institute, Vanderbilt University Medical Center, Nashville, Tennessee, United States of America; 2 Department of Radiation Oncology, Vanderbilt University Medical Center, Nashville, Tennessee, United States of America; 3 Department of Ophthalmology, Emory University School of Medicine, Atlanta, Georgia, United States of America; The University of Hong Kong, Hong Kong

## Abstract

**Background:**

Cumulative oxidative damage is implicated in the pathogenesis of age-related macular degeneration (AMD). Nuclear factor erythroid 2-related factor 2 (NRF2) is a transcription factor that plays key roles in retinal antioxidant and detoxification responses. The purposes of this study were to determine whether NRF2-deficient mice would develop AMD-like retinal pathology with aging and to explore the underlying mechanisms.

**Methods and Findings:**

Eyes of both wild type and *Nrf2^−/−^* mice were examined *in vivo* by fundus photography and electroretinography (ERG). Structural changes of the outer retina in aged animals were examined by light and electron microscopy, and immunofluorescence labeling. Our results showed that *Nrf2^−/−^* mice developed age-dependent degenerative pathology in the retinal pigment epithelium (RPE). Drusen-like deposits, accumulation of lipofuscin, spontaneous choroidal neovascularization (CNV) and sub-RPE deposition of inflammatory proteins were present in *Nrf2^−/−^* mice after 12 months. Accumulation of autophagy-related vacuoles and multivesicular bodies was identified by electron microcopy both within the RPE and in Bruch's membrane of aged *Nrf2^−/−^* mice.

**Conclusions:**

Our data suggest that disruption of *Nfe2l2* gene increased the vulnerability of outer retina to age-related degeneration. NRF2-deficient mice developed ocular pathology similar to cardinal features of human AMD and deregulated autophagy is likely a mechanistic link between oxidative injury and inflammation. The *Nrf2^−/−^* mice can provide a novel model for mechanistic and translational research on AMD.

## Introduction

AMD is the leading cause of severe visual impairment in elderly Americans, with an estimated 1.75 million people having advanced forms of the disease [1], [2]. A key pathological feature of AMD is age-dependent, progressive degeneration of the outer retina including the RPE, Bruch's membrane (BrM) and the underlying choroid [3], [4]. The pathogenesis of AMD likely involves multiple genetic, environmental, and demographic factors. Although major genetic variations of AMD have been identified in recent years [5], [6], their biological functions remain largely elusive. Similar to other complex human diseases, the influence from minor risk alleles and gene-environment interactions with other risk factors, such as advanced age and oxidative stress, can be critical in defining the individual course of AMD initiation, progression and therapeutic responses [7], [8].

NRF2 is a master regulator of endogenous antioxidant protection and is commonly involved in the transcriptional control of phase II detoxification enzymes [9]. It heterodimerizes with small Maf proteins and binds to the *cis*-acting antioxidant response element (ARE) sequence in the promoter regions of phase II genes [10]. Instead of relying on any single antioxidant enzyme, NRF2 activation leads to a concerted upregulation of a battery of protective proteins with coordinated functions at different steps of the detoxification process. *Nrf2* knockout mice have normal embryonic development and their basal level of antioxidant status in many tissues is not different from wild type mice [11]. However, the *Nrf2^−/−^* mice have increased sensitivity to a variety of pharmacological and environmental toxicants [12], [13]. NRF2 is also an important regulator of microglial function [14] and chronic neuroinflammation [15]. NRF2-deficient mice have been reported to exhibit more astrogliosis and microgliosis [16].

Several mouse models of AMD have been established by disrupting the balance between oxidative stress and antioxidant protection. Mice deficient of key antioxidant enzymes, either SOD1 or SOD2, developed age-dependent degeneration of the retina with certain phenotypes resembling AMD [17], [18]. Immunizing mice with an oxidation fragment of docosahexaenoic acid (DHA), carboxyethylpyrrole (CEP), resulted in autoimmune responses and dry AMD-like lesions in the retina [19]. A recent study reported that albino rats exposed to intense cyclic light developed photoreceptor damage and CNV in a relatively short time frame [20]. While many of the AMD-like phenotypes can be recapitulated by these models, their experimental approaches were mainly to overwhelm the retinal antioxidant system by exceedingly high levels of oxidant signals. How RPE cells utilize their elaborate endogenous protective mechanisms to repair and recover from oxidative injury is often overlooked.

To better understand the endogenous protective mechanisms that are involved in the different stages of RPE/choroid degeneration and CNV development, we studied the age-dependent retinal pathology in *Nrf2* knockout mice. Our data showed that *Nrf2^−/−^* mice developed age-dependent degeneration of the RPE and choriocapillaris, spontaneous CNV and deposits of inflammatory proteins in the sub-RPE space. Each of these features is observed in human AMD eyes, suggesting that the *Nrf2^−/−^* mouse can be a useful tool for probing specific aspects of the disease mechanisms.

## Results

### Clinical Examination of Age-Related Phenotype in Nrf2^−/−^ Mice

**Figure 1:**
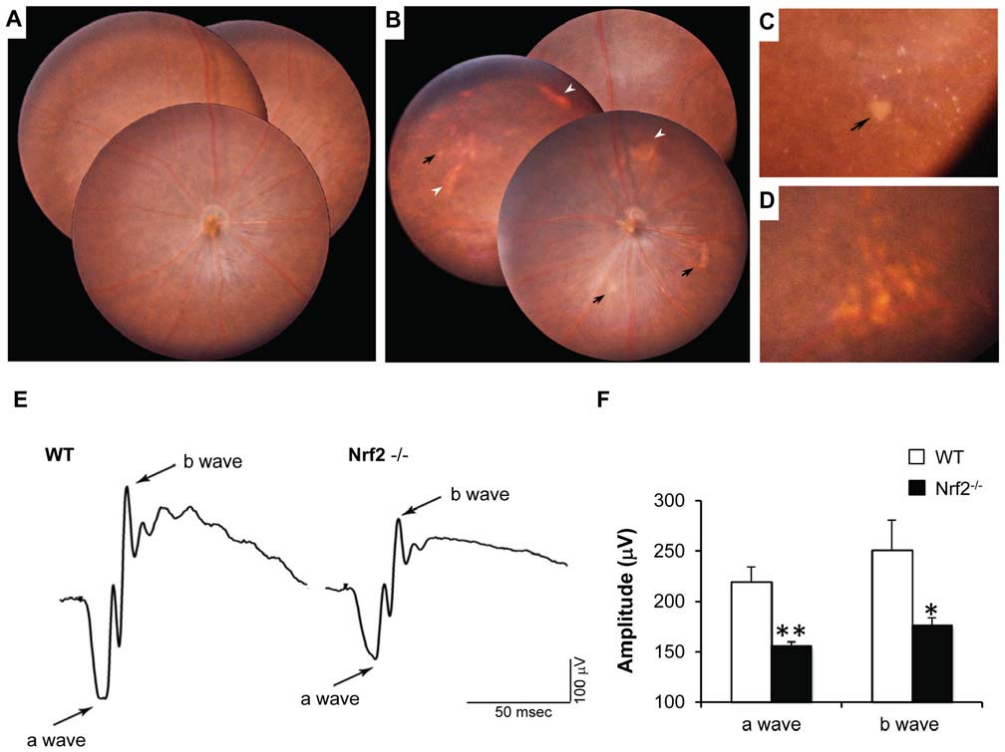
Early AMD-like degeneration in *Nrf2^−/−^* mice. (A) Normal fundus photograph from a 12-month-old wild-type mouse. (B) Merged photos from central and peripheral retina from a 12-month-old knockout mouse, showing both dotted and patchy deposits (arrows) and RPE mottling (arrowheads). (C) Magnified picture showing spots with sharp outline and a large patchy deposit in the mid-peripheral retina (arrow). (D) Soft drusen-like deposits with larger size and ambiguous border. (E and F) Scotopic ERG recordings at +10 dB (25 cd·s/m^2^) flash intensity, showing significantly decreased a- and b-wave amplitudes in *Nrf2* ^−/−^ mice when compared to age-matched wild-type mice (n = 6 per group; *P<0.05, ** *P*<0.01, Student's t-test).

Drusen are hallmark lesions of AMD [4]. We performed ocular funduscopic examination of 30 Nrf2^−/−^ mice (2–18 months) and 12 age-matched wild-type mice for drusen-like deposits in the retina (Table 1). The fundi of wild-type mice were normal at all age groups examined (Fig. 1A). Nrf2^−/−^ mice showed normal fundi before 8 months of age (n = 5) and were indistinguishable from aged-matched wild-type mice (n = 3). Between 8 to 11 months, Nrf2^−/−^ mice (n = 5) started to grow small, dome-shaped hard drusen with sharp borders and whitish color (Fig. 1C). Between 11 to 18 months, knockout mice (n = 20) presented more soft drusen-like deposits with larger size, yellowish color and ill-defined borders, as well as atrophic lesions of RPE mottling in the mid-peripheral retina (Fig. 1B and 1D).

**Table 1:** Summary of Funduscopic, Histological findings in *Nrf2^−/−^* mice (incidences/eyes examined).

	*Nrf2^−/−^* mice		RPE Pathology			Sub-RPE Deposit		Other	
Histology	Age (months)	No. of Animals	Hypo-pigmentation	Hyper-pigmentation	Vacuole	Drusen	Diffused Elevation	Subretinal Cells	CNV
	1–7	8	1/11	1/11	0/11	0/11	0/11	2/11	0/11
	11–13	9	5/12	9/12	8/12	2/12	10/12	8/12	1/12
	14–17	3	2/5	3/5	5/5	1/5	4/5	3/5	2/5
Fundus examination	Age (months)	No. of Animals	RPE mottling			Nodular deposits		Patchy deposits	
	2–7	5	2/9			0/9		0/9	
	8–11	5	7/10			7/10		1/10	
	11–18	20	30/35			27/35		20/35	

The in vivo visual function of wild type and Nrf2 knockout mice were evaluated by scotopic ERG. At 6 months, no significant difference was observed between Nrf2^−/−^ and control mice (data not shown). However, moderate but significant decreases of both a- and b-wave amplitudes were detected in 12-month-old Nrf2^−/−^ mice (Fig. 1E).

### RPE Degeneration and CNV in Nrf2^−/−^ Mice

**Figure 2:**
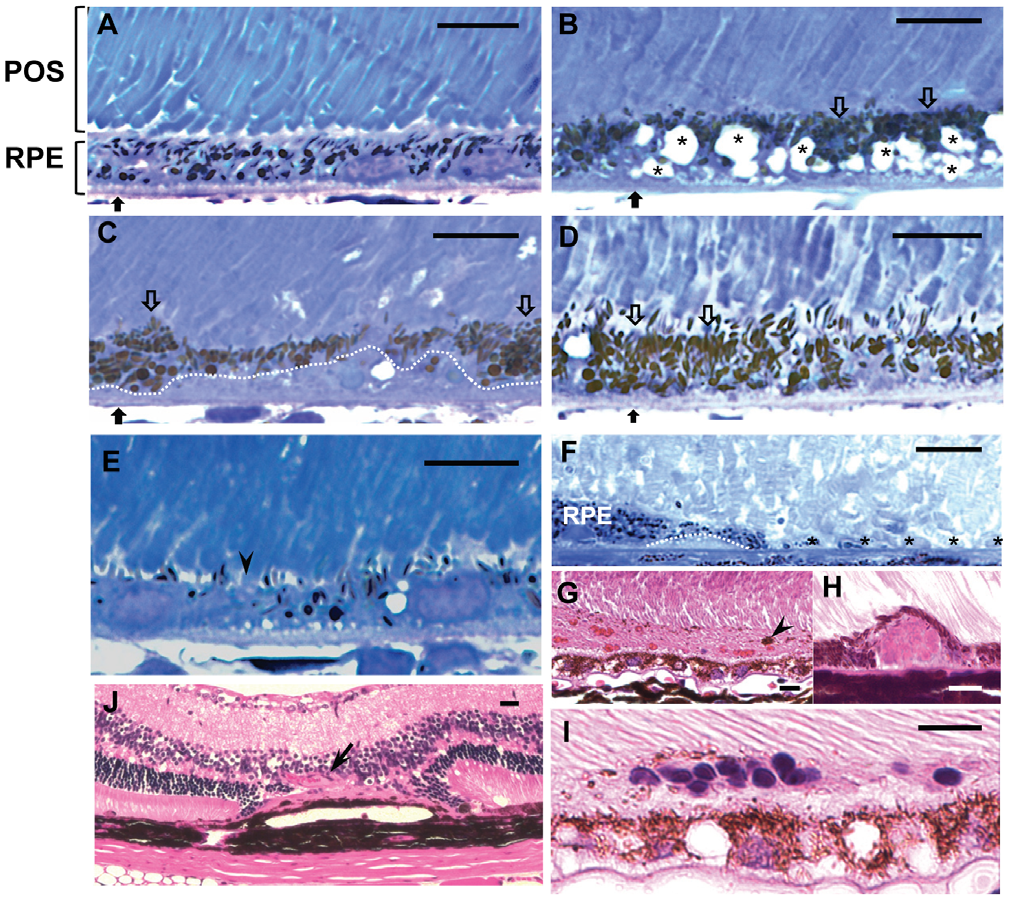
Histopathology of RPE degeneration and CNV in *Nrf2^−/−^* mice. (A) Normal retina from a 12-month-old wild-type mouse, on toluidine blue-stained 1 µm plastic section. (B–F) Representative degenerative pathology in 12 month-old *Nrf2^−/−^* mice, including RPE vacuolation (B, asterisks) and BrM thickening (B, black arrows) hyperpigmentation (B to D, open arrows), hypopigmentation (E, arrowhead), sub-RPE deposits (C and F, under the dotted line) and loss of RPE cells (F, asterisks). (G–I) H&E stained-paraffin sections, showing (H) dome-shaped drusen deposit, (I) subretinal cell infiltration, (G) subretinal hemorrhage with melanin containing cells (arrowhead), (J) choroidal neovascularization through compromised BrM into retina (arrow). (Scale bars: A–I  = 10 µm; J  = 20 µm)

Histopathologic examination was performed on 20 Nrf2 knockout mice and 15 age-matched wild-type controls (4–17 months) by light microscopy (Table 1). Wild-type mice showed normal retina at all ages examined (Fig. 2A). In contrast, age-dependent degenerative changes were observed in the outer retina of Nrf2^−/−^ mice by end of the first year. On toluidine blue-stained slides, signs of RPE degeneration, including extensive vacuolation (Fig. 2B), hyperpigmentation (Fig. 2B–2D), hypopigmentation (Fig. 2E) and occasional areas with complete loss of RPE (Fig. 2F), were identified in knockout mice at 12 months of age. Areas of continuous basal deposition underneath the RPE were evident in 11 of the 12 Nrf2^−/−^ mice examined at advanced age (Fig. 2C and Table 1), but were rarely detected in age-matched wild-type controls (n = 1/10, P<0.001, Fisher's Exact Test). On hematoxylin and eosin (H&E)-stained sections, drusen were detected as deposition of dome-shaped extracellular material between the BrM and RPE (Fig. 2H). Subretinal cellular infiltrates could be occasionally seen near the RPE lesions (Fig. 2I).

CNV is a characteristic feature of exudative AMD. Through histological examination, we observed spontaneous CNV development in 3 out of 17 eyes from Nrf2^−/−^ mice between 11 and 17 months (Table 1). At the site of CNV, there was focal RPE hyperplasia and atrophy of overlying photoreceptors and outer nuclear layer (Fig. 2J). Subretinal hemorrhage and exudate (Fig. 2G), which are two reliable ophthalmologic signs of CNV [21], were also present in eyes with CNV. The percentage of Nrf2^−/−^ mice developing spontaneous CNV was similar to what has been reported in aged ApoE knockout mice fed with high fat diet [22].

**Figure 3:**
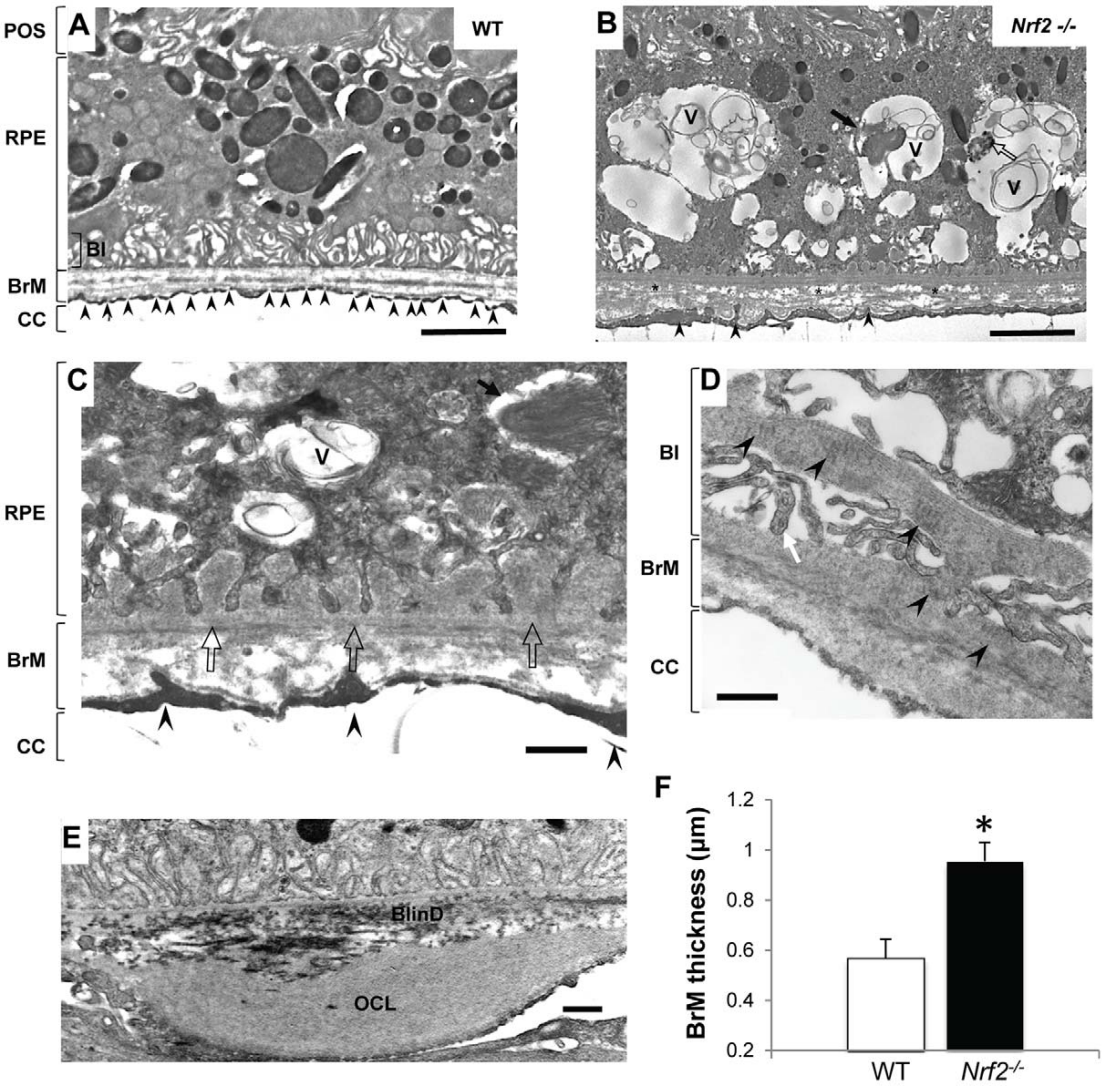
Ultrastructual changes in outer retina of aged *Nrf2^−/−^* mice. (A) Electron micrograph of a 12-month-old wild-type mouse. Endothelial fenestrations of choriocapillaris (CC) were marked by arrowheads. (B) RPE of an *Nrf2^−/−^* mouse at 12-months showed large vacuoles (V) containing membranous debris, undigested POS (arrow) and melanin-containing materials (open arrow). BrM was thickened with disorganized collagen and elastin fibers (asterisks). (C) Basal infoldings were replaced by continuous basal deposits (open arrow). (D) Higher magnification showing basal laminar deposits and traverse-banded structure (arrowhead). (E) Extensively thickened outer collagenous layer (OCL) and basal linear deposits (BlinD). (F) Increased BrM thickness in aged *Nrf2^−/−^* mice. Data presented are average of measurements from 6 mice per group (mean ± SE) (* P<0.01, Student's t-test).

When examined by transmission electron microscopy (TEM), wild-type mice showed normal structure of RPE, BrM and choriocapillaris with well-developed RPE basal infolding and endothelial fenestration (Fig. 3A). In Nrf2^−/−^ mice, degenerative changes of the RPE were apparent at 12 months. The RPE cells were highly vacuolated with membranous debris (Fig. 3B). Areas of basal infoldings were replaced by amorphous and homogenous material deposits (Fig. 3C), which were similar to continuous basal laminar deposits (BlamD) found in human AMD eyes [23]. Some deposits included banded structures (Fig. 3D) that resembled the long-spaced collagen found in BlamD of human AMD [24].

Compared to age matched wild-type controls, the BrM was significantly thickened in Nrf2^−/−^ mice at 12 months of age (Fig. 3A and 3B). The mean thickness of BrM was 0.955±0.065 and 0.569±0.075 µm in knockout and wild-type mice (mean±SEM), respectively (Fig. 3F) (P<0.01, unpaired t-test, n = 4 for each strain). The thickening was generally in a diffuse pattern with disrupted collagen fibers observed in the inner collagenous and elastin layer of the BrM (Fig. 3B), which is also characteristic of AMD [24]. In some areas, we observed extensive thickening of the outer collagenous layer of the BrM, often accompanied with granular debris both inside the RPE and the BrM (Fig. 3E). In addition, electron-dense debris was identified accumulating between the RPE basement membrane and the elastin layer of BrM, resembling basal linear deposits (BlinD) in human AMD (Fig. 3E).

As part of the blood-retina barrier [25], the choroidal endothelial cells in wild-type retina were highly fenestrated and abutting on the BrM (Fig. 3A). In NRF2-deficient mice, however, fenestrations were significantly lost with obvious thickening of the choriocapillary endothelium (Fig. 3B and 3C). In some areas, the endothelial processes broke through the basement membrane and protruded into the BrM (Fig. 3B and 3C), which could mark the initiation of abnormal growth of blood vessels in these areas [26], [27], [28]. Taken together, the EM and histopathology data demonstrated that Nrf2^−/−^ mice developed AMD-like degeneration of RPE, BrM and choriocapillaris, as well as spontaneous CNV at advanced age.

### Lipofuscin Accumulation and Sub-RPE Deposition of Inflammatory Proteins in Nrf2^-/-^ Mice

**Figure 4:**
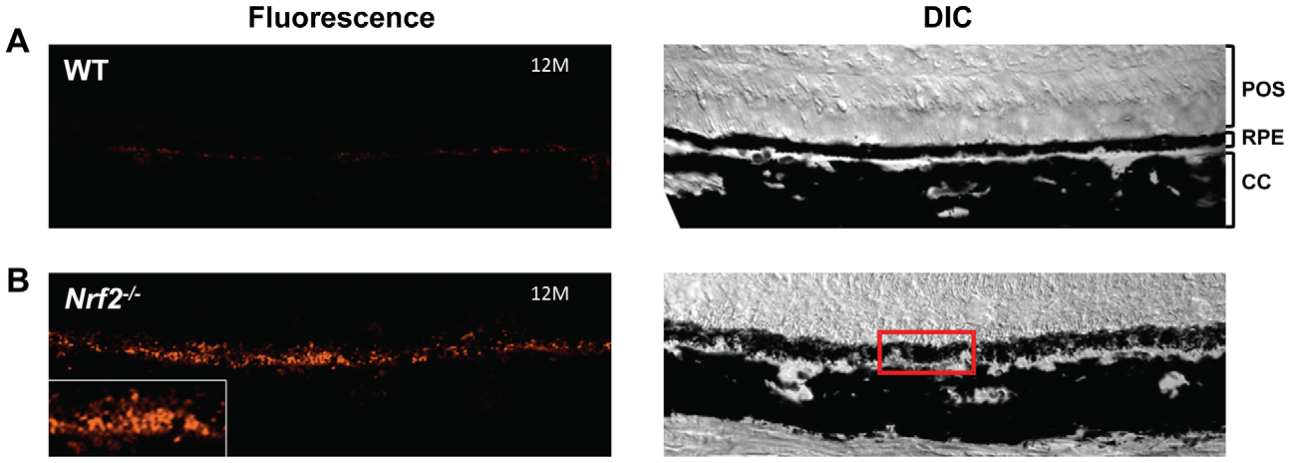
Lipofuscin accumulation in *Nrf2^−/−^* mice. RPE autofluorescence was measured in WT (A) and *Nrf2^−/−^* mice (B) at 12 months. The insert in (B) is magnified from an area of RPE clumps as marked by the red box. (Scale bar, 100 µm)

Age-dependent accumulation of lipofuscin and the resulting autofluorescence in human eyes are associated with RPE atrophy and progression to advanced AMD [22], [29], [30], [31], [32]. In Nrf2^−/−^ mice, we observed the accumulation of autofluorescent granules in RPE cells in an age-dependent manner (Fig. 4). Eyes from wild type mice showed only very dim autofluorescent materials present underneath the RPE at 12 months. In Nrf2^−/−^ mice at the same age, however, the RPE autofluorescence was much more prominent (Fig. 4). Noticeably that although diffusely dispersed in the RPE layer, lipofuscin-like particles were prone to aggregate around cells of compromised integrity.

**Figure 5:**
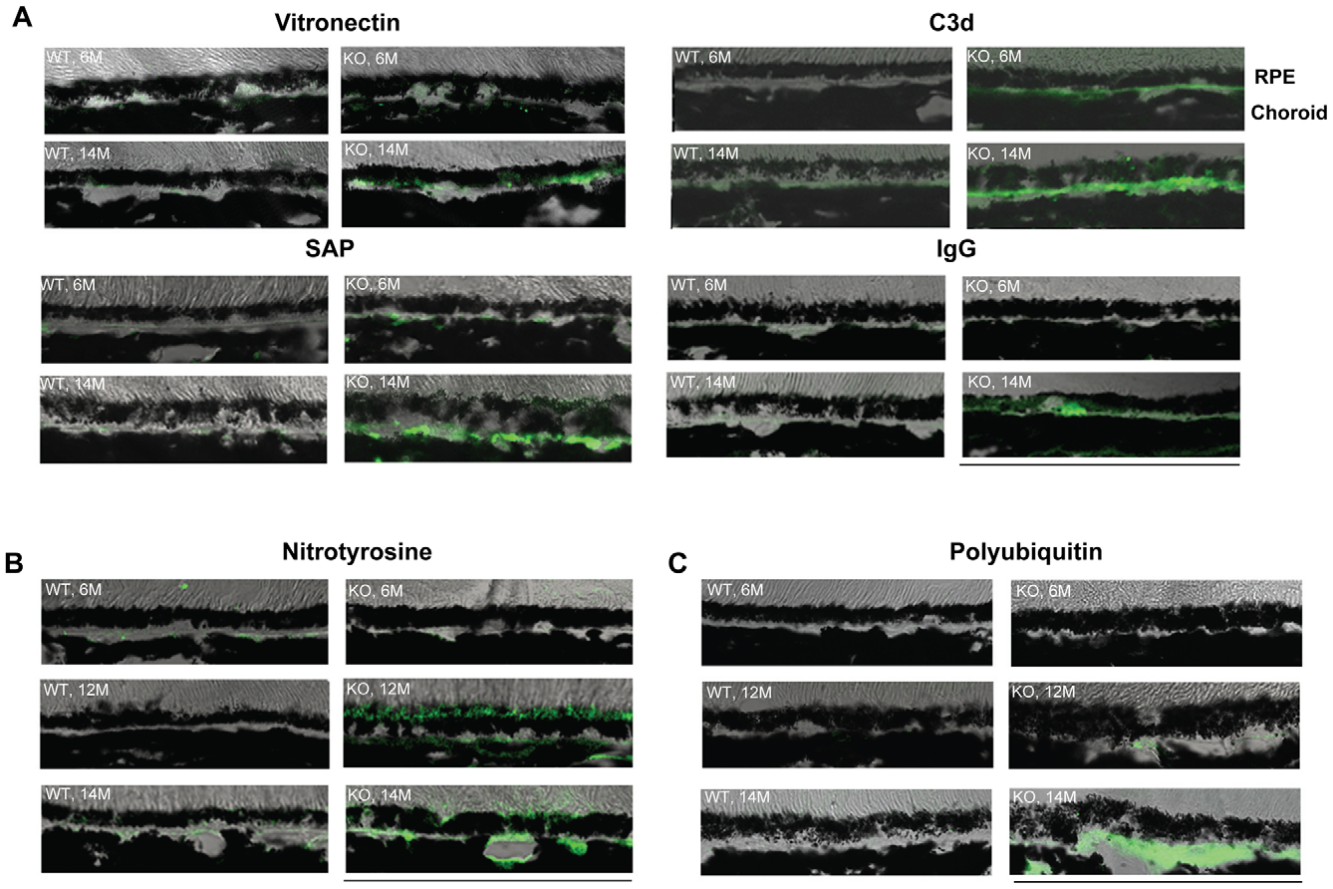
Immunofluorescence staining of sections of outer retina from WT and *Nrf2^−/−^* mice. Cryosections were prepared from 6, 12 and 14 month-old mice and stained with indicated antibodies. Images were acquired by confocal microscopy using identical setting between WT and *Nrf2^−/−^* mice. (Scale bar, 100 µm)

Human drusen have been reported to contain components of complement system and extracellular matrix [5], [33], [34]. In NRF2-deficient mice, we observed age-dependent increase of immunoreactivity of C3d, serum amyloid P (SAP), vitronectin, and immunogloubin (IgG) in the RPE and BrM (Fig. 5A). Staining of 3-nitrotyrosine, a marker of oxidatively damaged proteins, also showed age-dependent increase in RPE and sub-RPE space (Fig. 5B). Nitrosylated proteins were mainly located at the apical side of the RPE at 12 month; however, they tended to be redistributed towards the BrM at 14 months (Fig. 5B).

### Deregulated Lysosome-Dependent Degradation Pathway in Nrf2^−/−^ Mice

**Figure 6:**
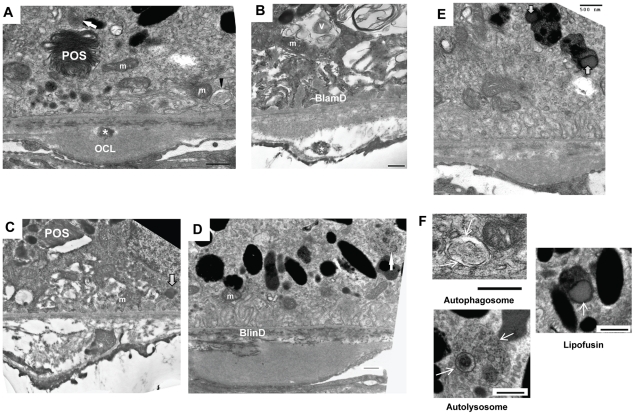
Accumulation of intermediate structures of lysosome-dependent degradation pathways in *Nrf2^−/−^* mice. At sites of BrM with abnormalities, RPE cells showed increased presence of autophagosome (A, arrowhead and F) and autolysosome (A, D and F, arrow), swollen mitochondria fragments next to autophagic vacuoles (A and B), undigested POS (A and C), and lipofuscin (C and E, open arrow, and F). Heterogeneous electron-dense deposits were detected in BrM as well (asterisks). OCL, Outer collagenous layer; m, mitochondria; V, Vacuole; Scale bars: 500 nm.

A major function of the RPE is the constant removal of photoreceptor outer segments (POS) via phagocytosis. As shown in Fig. 6, Nrf2^−/−^ mice had accumulation of undigested POS (Fig. 6A and 6C), indicating degenerated RPE cells became less efficient in lysosome-mediated organelle turnover. Adult RPE cells are considered postmitotic and utilizing autophagy, a lysosome-dependent self-renewal process, to remove damaged macromolecules and organelles [35], [36]. With aging, Nrf2^−/−^ RPE showed signs of deregulated autophagy. Intermediate structures of autophagy, such as autophagosome and autolysosome, were readily detectable by EM (Fig. 6A, 6D and 6F). Swollen mitochondria were often found in close proximity to autophagy vacuoles (Fig. 6A and 6B), which were indicative of awry autophagy of mitochondria (mitophagy) [37]. Areas of increased presence of lipofuscin were also detected (Fig. 6E). Notably the accumulation of undigested intermediates of phagocytosis and autophagy were often present near the site of BrM abnormalities. Electron dense structures, reminiscent of autophagy-related vacuoles, appeared to have directional movement from the RPE into the choriocapillaris (Fig. 6A–C). Accordingly, poly-ubiquitinated protein aggregates accumulated underneath the RPE with aging (Fig. 5C). These data collectively suggest that Nrf2^−/−^ RPE had defects in lysosome-dependent degradation and were less efficient in removal of oxidatively damaged protein aggregates and organelles by autophagy to achieve cellular homeostasis.

## Discussion

In the present study we demonstrated that mice deficient in NRF2 displayed many of the cardinal pathological features of human AMD, including drusen deposition (Fig. 1), age-related degeneration of RPE, BrM and choriocapillaris (Fig. 2 and Fig. 3), increased RPE autofluorescence (Fig. 4) and development of spontaneous CNV (Fig. 2). Compared to age-matched wild-type mice, *Nrf2^−/−^* mice showed moderate decrease of a- and b-wave amplitudes on ERG (Fig. 1). Similar findings of decreased rod-driven ERG response have been reported previously in patients with AMD [38], [39]. The deposition of IgG and components of complement pathway as well as its regulators (Fig. 5) were similar to what have been found in human AMD eyes [34], [40], [41], [42]. The median lifespan of *Nrf2* knockout mice was reported to be 106 weeks [43]. Therefore, the retinal phenotype of degeneration occurred during the last 1/3 of their life span. Because we did not perform continuous sections of the whole eye specimen, the incidence of CNV and other focal lesions (Table 1) could have been underestimated. Taken together, the results from our studies support the causative role of oxidative stress in the pathogenesis of AMD and suggest that *Nrf2^−/−^* mice represent a new animal model of AMD.

Oxidative retinal injury and AMD-related pathology have been demonstrated in SOD1 and SOD2-deficient mice [17], [18]. Those animals developed progressive degeneration of the whole retina. Severe loss of photoreceptor cells occurred before or at the same time as RPE degeneration and such time course is not typical of human AMD. In addition to the different phenotype displayed by *Nrf2* and *SOD* knockout mice, these proteins have distinct antioxidant functions. While SODs are responsible for the constitutive removal of reactive intermediates generated from normal metabolic processes, NRF2 is mostly activated by signaling mechanisms that change the cellular thiol/disulfide redox status [9]. Consequently, the pathology of *SOD1^−/−^* mice developed at much earlier time point and progressed as a linear function of age [18]. In contrast, the degeneration of RPE/BrM/Choroid in NRF2-deficient mice occurred mainly at the last 1/3 of their life span and appeared to progress exponentially as a function of age, which is typical for age-related degenerative diseases. In the future, it will be interesting to learn whether other genetic or environmental factors can interact with the NRF2 system and change the disease course in these animals.

Increased presence of autophagic vacuoles is an initial sign of deregulated autophagy [44]. EM studies of aged RPE in NRF2-deficient mice showed accumulation of intermediate structures of autophagy, undigested POS, lipofuscin and abnormal mitochondria in close proximity to autophagosome and vacuoles (Fig. 6). Autophagy is a conserved lysosomal pathway which is essential for organelle turnover and removal of aggregated proteins [45]. During autophagy, unwanted proteins and organelles are sorted to double-membraned autophagosomes (Fig. 6F), which are further delivered and fused with lysosomes to degrade sequestered cargos and eventually recycle the generated macromolecules as catabolic substrates. A unique feature of the RPE cells is the phagocytosis of POS which generate reactive products such as A2E (N-retinylidene-N-retinylethanolamine), which is a potent inhibitor of lysosomal function [46]. Deregulated autophagy has been associated with various neurodegenerative diseases such as Alzheimer [47], Huntington's [48] and Parkinson's disease [49]. It is likely that autophagy is also a central mechanism protecting against AMD-related degenerative changes in the RPE.

Similar to the EM observations from *Nrf2^−/−^* mice, a previous report showed that autophagosome-like structures accumulated in the RPE of human AMD eyes [50]. The accumulation of various intermediate forms of autophagic vacuoles and multivesicular bodies could be due to either increased autophagic flux or decreased final degradation by lysosome. In NRF2-deficient RPE, the compromised antioxidant system may not be sufficient to protect the lysosomes from injury caused by POS-derived reactive intermediates when the animals age. On the other hand, several recent publications showed that NRF2 can regulate the expression of p62 [51], [52], which is a receptor protein that mediates the cargo assembly during the initial formation of autophagic vesicles [53]. Although we confirmed the similar findings in cultured RPE cells, no *in vivo* change of p62 mRNA was found in the RPE of *Nrf2^−/−^* mice (data not shown). How NRF2 directly regulates autophagy pathways in the RPE remains to be characterized by future studies.

There is a plethora of evidence supporting the hypothesis that innate immune response plays an active role in AMD pathogenesis [5], [54]. In our model, we observed the sub-RPE deposition of complement fragment C3d, the end degradation product of C3, as well as vitronectin and serum amyloid P, which are regulators of complement pathways. The data suggest that inflammation might contribute to the phenotype of the *Nrf2^−/−^* mice. The deposit of the immunoreactive proteins in the sub-RPE space may be related to autophagy. RPE cells with impaired lysosomal function and autophagic activity may release cellular metabolic waste in membrane-enclosed vesicular bodies via exocytosis (Fig. 6A to 6C). Deposit of the polyubiquitinated materials in the sub-RPE space and BrM (Fig. 5) may further lead to drusen formation and initiate innate immune responses involving complement activation. Consistently, a recent study by Wang et al. showed positive staining of exosome markers CD63 and CD81 in human AMD eyes [50].

**Figure 7:**
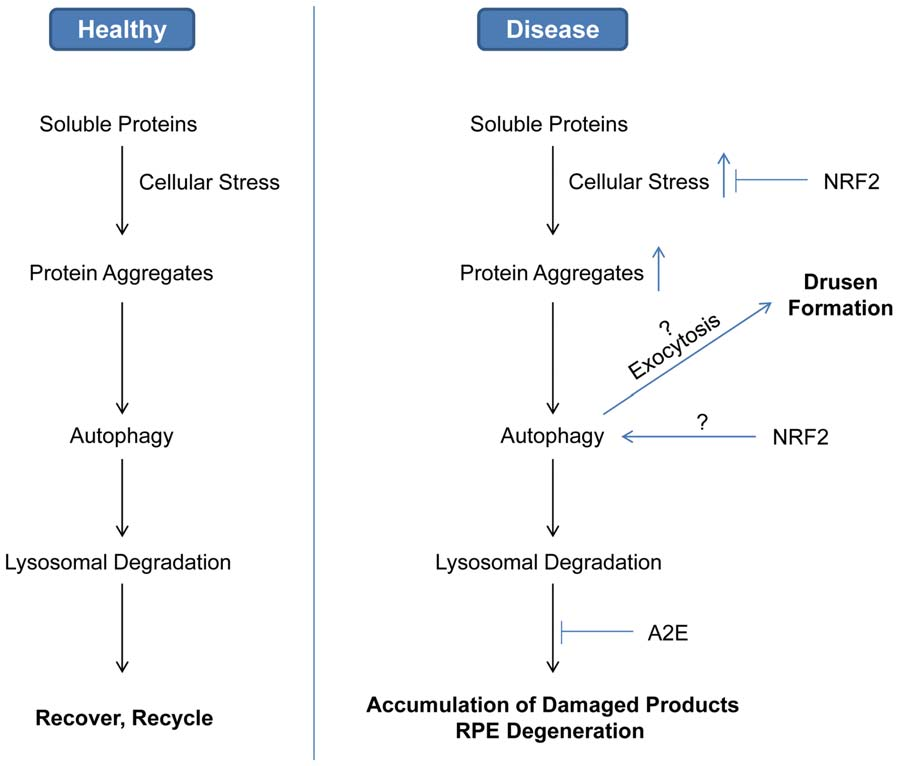
Schematic model integrating oxidative stress, autophagy and lysosomal function into the etiology of AMD. RPE cells are exposed to high levels of cellular stress and, when healthy, damaged proteins and organelles are removed promptly by autophagy. Under disease conditions, such as decreased antioxidant defense and lysosome inhibition, self-renewal by autophagy becomes less efficient. The resulted cellular waste products can be exported by exocytosis and contribute to sub-RPE deposit and drusen formation. NRF2 can be involved in regulating both the antioxidant responses and the autophagic activities.

Based on our experimental data and literature report, we propose a model of the roles of autophagy in AMD (Fig. 7). In normal RPE cells, autophagy is responsible for removing aggregates of polyubiquitinated proteins that cannot be processed by proteasomes. Cargos inside autophagosomes will be targeted to lysosome for degradation and recycled for catabolism. Under conditions predisposing to age-related RPE degeneration, elevated cellular stress will cause increased damage to proteins and organelles and increased burden of autophagy. Reactive metabolites such as A2E can inhibit lysosome-mediated turn over and lead to accumulation of waste materials eventually overwhelming the capacity of autophagy. Consequently, the undigested proteins could be exported into extracellular space and BrM via exocytosis, and promote drusen formation and local inflammation.

NRF2 has many other documented functions. It can regulate neuroinflammation. MPTP treatment of *Nrf2^−/−^* mice caused more pronounced activation of astrocytes and microglia than the wild type mice received the same treatment [16]. Cultured *Nrf2^−/−^* microglia and astrocytes also showed higher expression of pro-inflammatory genes, such as IL-6, TNF-α, IL-β and iNOS [16]. However, we did not observe abnormal GFAP staining of *Nrf2^−/−^* retina and did not find significant differences in retinal IL-6 and IL-1β expression (data not shown). NRF2 can regulate mitochondrial antioxidant function [55]. Mitochondrial glutathione content, as well as MnSOD and catalase activities, can be elevated by sulforaphane treatment [56]. NRF2 can be involved in redox regulation of mitochondrial permeability transition [55] and, therefore, can be important in protecting RPE cells from oxidant-induced apoptosis. NRF2 may also regulate longevity. Long-lived Snell dwarf mice had increased tissue expression of metallothionein 1, heme oxygenase-1, glutamate cysteine ligase and thioredoxin reductase, all of which function downstream of NRF2 [57]. On the other hand, caloric restriction could not extend the life span of *Nrf2^−/−^* mice [43]. Aging is a primary demographic factor of AMD. All of these NRF2-mediated signaling mechanisms may contribute to the protection by NRF2 on RPE aging and age-related degeneration.

In summary, our study demonstrated that mice deficient in NRF2 presented retinal pathology of age-related drusen formation, RPE/BrM degeneration, sub-RPE deposition of inflammatory proteins and spontaneous CNV, all of which are key features of human AMD. Our model provides a novel platform for future research on mechanisms of gene/environment interaction in the etiology of AMD; and can be further optimized for pre-clinical drug screening of interventional agents against both dry and exudative AMD.

## Materials and Methods

### Animals

Protocols for animal breeding, housing and handling were approved by the Vanderbilt Institutional Animal Care and Use Committee (IACUC) (Protocol number M/09/159). All procedures were conducted in accordance with the ARVO statement for the Use of Animals in Ophthalmic and Vision Research. The *Nrf2^−/−^* mice [58] were kindly provided by Dr. J.Y. Chan at University of California, Irvine. Exons 4 and 5 of the mouse *Nfe2l2* gene, which encodes the basic leucin zipper domain that controls transcriptional activation, was replaced by a LacZ reporter gene [58]. Homozygous *Nrf2^−/−^* mice had hybrid genetic background of C57BL/SV129. Mouse breeding and genotyping were performed following published methods [59]. *Nrf2^−/−^* mice have normal embryonic development [58] and normal growth rate at young age [59], although the average litter size is only about 60% of wild type breeding mice. Mice were housed at pathogen-free facilities of Vanderbilt Division of Animal Care facilities, and were kept on diurnal cycles of 12 h light and 12 h dark with *ad libitum* access to food and water.

### Fundus Photography

A modified otoscope system was used for mouse funduscopic examination, according to methods described in the literature [60], [61]. Animals were anesthetized by intraperitoneal injection of ketamine and xylazine, and pupils were dilated by topical administration of 1% tropicamide. An endoscope (1218AA; Karl Storz) attached to an objective lens (Nikkor AF35/f1.8, Nikon) and a reflex digital camera (D90; Nikon) was used to take digital fundus photographs of central, nasal and temporal retina.

### Electroretinography (ERG)

Scotopic ERG was recorded using an UTAS-E3000 rodent ERG system (LKC Technologies). Mice were dark-adapted for at least 12 hours and anesthetized by ketamine/xylazine. Topical administration of 1% tropicamide and 0.4% oxybuprocaine was used to dilate the pupils and depress the cornea reflex. ERG responses were evoked with a three-step protocol (0, +10 and 20 dB light flashes) and recorded with a DTL silver electrode (Diagnosys). The ERG Data were analyzed with EMWIN 8.1.1 software (LKC Technologies).

### Immunohistochemistry and Fluorescence Microscopy

Mice were terminally anesthetized and subjected to whole body perfusion with 4% paraformaldehyde in phosphate-buffered saline (PBS). Whole eyes were enucleated and post-fixed in the same fixative overnight before embedded in Tissue-TeK Cryomold (Electron Microscopy Sciences). Sagittal cryosections of 8 µm thickness were prepared from cornea to optic nerve and stained for various antigens of interest. To block nonspecific binding, tissue sections were incubated with normal serum appropriate to the secondary antibody species diluted in 0.5% Triton X-100/PBS. They were then incubated with primary antibodies followed by staining with Alexa Fluor®-conjugated secondary antibodies (Invitrogen). Fluorescence images were acquired by confocal microscopy (Carl Zeiss). Primary antibodies used for the study included anti-C3d, Nitrotyrosine, Vitronectin (R & D Systems), polyubiquitin (FK1, Enzo,) and Serum Amyloid P (Santa Cruz). Isotope-matched IgG was used as a negative control for each experiment.

For detection of RPE autofluorescence, freshly-cut frozen sections were air dried at room temperature for 2 hours before sealed with Fluoro-gel (Electron Microscopy Sciences). Confocal images were acquired by an FV 1000 system (Olympus) using excitation 543 nm and emission 570 nm [46], [62]. Images were taken from both wild type and knockout strains using identical settings to ensure comparable results.

### Histology and Electron Microscopy (EM)

Eyes from wild type and knockout mice at different ages were enucleated and post-fixed in 4% formaldehyde for 24 h before embedded in paraffin. Sagittal sections of 5 µm thickness were cut from cornea to optic nerve and stained with H&E. At least 10 slides from each eye were examined. If lesions were found in the outer retina, serial sections would be cut through the entire depth of the lesions.

For EM, mice were perfused with 2.5% glutaraldehyde in cacodylate buffer (0.1 M, pH 7.4) through left ventricle. Eyes were enucleated and fixed in the same buffer for 12 h at room temperature. Samples were then sent to either the Vanderbilt Cell Imaging Core or the L.F. Montgomery Laboratory of Ophthalmic Pathology at Emory Eye Center for post-fixation, dehydration and embedding in epoxy resin. Semi-thin sections (1 µm) through the optic nerve were prepared, stained with toluidine blue and examined by light microscopy. Ultrathin sections (0.5 µm) of selected areas were then prepared and stained with uranyl acetate and lead citrate for EM (CM-12 TEM; Philips). To measure the thickness of BrM, at least 10 digital images were captured for each sample at a magnification of 19,000X. A transparent grid was superimposed onto the micrograph, with RPE basement membrane aligned with the horizontal line. Five random measurements (altogether 50 measurements per sample) were made on each digital image using ImageJ software (http://imagej.nih.gov/ij). Areas with considerably thickened outer collagenous layer (Fig. 3E) were excluded. The thickness of BrM was determined by averaging all measurements of each group.

### Statistical Analyses

Data from two groups of animals were presented as means ± SEM, and Student's t-test was performed to analyze the difference of BrM. Fisher's exact test was employed to analyze the difference of histological events. Statistically significant was considered as *P* values <0.05.
